# Relationship Between Growth Mindset and English Language Performance Among Chinese EFL University Students: The Mediating Roles of Grit and Foreign Language Enjoyment

**DOI:** 10.3389/fpsyg.2022.935506

**Published:** 2022-07-07

**Authors:** Xiaoyu Hu, Gurnam Kaur Sidhu, Xin Lu

**Affiliations:** Faculty of Education, Languages and Psychology, SEGi University, Petaling Jaya, Malaysia

**Keywords:** growth mindset, grit, foreign language enjoyment, English language performance, language learning, positive psychology

## Abstract

There is no denying that there is ample evidence of numerous factors that influence language learners' success. Recently, there is a critical call to embrace positive psychology that is more open and appreciative of the positive influences in learning English as a foreign language (EFL). Set against this burgeoning area of study in language learning, this paper puts forward the findings of a study that aimed to examine the mediating roles of grit and foreign language enjoyment in the relationship between growth mindset and English language performance. The study employed a correlational research design involving 388 EFL students from one university in China. The data were collected through a questionnaire and an English language performance test. Using the structural equation modeling, this study found that the association between growth mindset and English language performance was partially mediated by grit and foreign language enjoyment. This indicates that students with a growth mindset tend to possess a higher level of grit as well as experience more enjoyment in learning English, which consequently can lead to students becoming more successful language learners. These findings provide significant implications for language teachers, educational material developers, and school administrators in China to embrace the affective domain postulated by positive psychology.

## Introduction

With the twin forces of globalization and internationalization, English is fast becoming a universal language. Against this background, there is an increasing demand for a workforce competent in English. Today in China, English as Foreign Language (EFL) is taught as a core subject at almost all levels of schools, including primary schools, high schools, and institutions of higher learning. Furthermore, English language performance (ELP) is viewed as one of the most important considerations in university admissions and job applications in China (Liu and Wang, 2021). Set against this significant prominence of English language acquisition, it has become a subject of much interest among researchers in China seeking to explore various influencing factors that have an impact on the EFL university students' ELP.

Among the amalgam of factors influencing ELP, positive psychology has recently emerged in the field of second language acquisition (SLA). Language teachers and learners could benefit greatly from positive psychology as they can alleviate the detrimental effects of negative aspects while harnessing the positive emotions in language learning and teaching (MacIntyre et al., 2016). To this discussion, Dewaele et al. (2019) added that positive psychology has inspired a critical call to investigate the positive attributes that can enhance language teachers' and learners' wellbeing. Wang Y. et al. (2021) highlighted that positive factors such as wellbeing, grit, resilience, enjoyment, and emotion regulation can impact learners' linguistic performance and progress. Such positivity often builds language learners' wellbeing and their motivation and desire to learn and succeed in foreign language achievement. Among numerous positive factors such as intelligence, personality, attention, interest, self-confidence, and motivation, three positive psychological and emotional factors that have gained growing attention in the field of SLA in recent years are growth mindset, grit and foreign language enjoyment (FL enjoyment).

Growth mindset is considered as a significant motivational variable that positively affects FL learning (Bai and Wang, 2020). Growth mindset refers to the belief that a person's intelligence and learning ability can be enhanced through hard work and dedication (Dweck, 2006). When learners possess a growth mindset, they are more inclined to have mastery-oriented objectives, which encourages them to persist even under adversity and hence can sustain interest in a task in spite of challenges (Tang et al., 2019). Growth mindset has been found to be positively associated with students' academic performance (Romero et al., 2010; Paunesku et al., 2011; Wang et al., 2020), as well as language learners' grit (Teimouri et al., 2020), motivation (Ryan and Mercer, 2012), enjoyment (Khajavy et al., 2021b; Wang H. et al., 2021), and language achievement (Rui and Muthikrishnan, 2019; Khajavy et al., 2021a,b). However, while the impact of growth mindset has been studied extensively across a wide range of academic fields, including psychology and education, it has received inadequate attention in FL learning (Lou and Noels, 2019), especially in highly competitive test-orientated learning environments, like China. Therefore, to further examine the role of growth mindset in language learning, this present study was conducted to explore the predictive mechanisms for growth mindset in connection with ELP. Specifically, this study aimed to examine the mediating roles of grit and FL enjoyment in the association between growth mindset and ELP.

Another much-discussed positive factor is grit. According to Duckworth et al. (2007), grit can be defined as the persistence and enthusiasm that individuals show to achieve higher goals. It is among the most critical individual characteristics for FL learning (Khajavy et al., 2021a). Previous studies have indicated that grit is positively correlated with students' academic achievement (Duckworth and Quinn, 2009; Akos and Kretchmar, 2017) and language learning success (Wei et al., 2019; Lee, 2020; Teimouri et al., 2020; Liu and Wang, 2021; Sudina and Plonsky, 2021). Nevertheless, although grit has lately piqued the interest of scholars in the field of language learning, there have been a limited number of studies in this field, and additional studies are needed to better understand how grit influences language learning (Wang Y. et al., 2021). This study sought to determine the mediating effects of grit on the link between growth mindset and ELP.

A growing body of research has recently focused on the vital link between learning enjoyment and learners' wellbeing. This interest has also edged into FL learning since the advent of positive psychology in SLA (Guo, 2021; Botes et al., in press). Enjoyment can be described as “a sense of novelty and accomplishment” (Csikszentmihalyi, 2008, p. 46). There has been repeated evidence that FL enjoyment is positively linked to language learners' academic performance (Dewaele and Alfawzan, 2018; Jin and Zhang, 2018; Li et al., 2020; Guo, 2021), willingness to communicate (WTC) (Khajavy et al., 2018), trait emotional intelligence (Li, 2019), and motivation (Zhang et al., 2020). Shao et al. (2020) believed that students who experienced more positive emotions such as enjoyment and pride in contrast to less negative emotions like anxiety, and boredom were more likely to succeed in language learning. However, it remained unknown if FL enjoyment could mediate the association between growth mindset and ELP, in the context of an exam-oriented social-cultural learning environment and this aspect was addressed in this study.

FL learning is an arduous task, especially when the exposure to the target language is very limited. This becomes even more critical in China and other Asian countries where the test-oriented education has been long practiced and scores and high-stakes testing are highly valued (Lee and Zhou, 2015; Guo et al., 2016). In such a challenging environment, to be a successful language learner, a student needs to embrace the belief that language learning ability can be enhanced through hard work and dedication (Mercer and Ryan, 2010; Ryan and Mercer, 2012), keep “perseverance and passion for long-term goals” (Duckworth et al., 2007, p. 1087), and develop positive emotions rather than negative ones (Dewaele and Alfawzan, 2018). It is noteworthy that these three positive qualities (i.e., growth mindset, grit, and FL enjoyment) are malleable, and can be cultivated through intervention and instruction (Dewaele et al., 2018; Lou and Noels, 2019; Teimouri et al., 2020). According to Wang Y. et al. (2021), language teachers, educational material developers, and school administrators can take necessary steps to foster language learners' growth mindset, grit, and FL enjoyment in order to prepare students for the fierce competition and hardships in FL learning.

In summary, the exploration of positive factors such as growth mindset, grit and FL enjoyment requires special attention in the Chinese EFL context. Understanding this crucial relationship between learning and learners' enjoyment and wellbeing is of significance because like most Asian countries where learning is often influenced by competitive and stress-fueled high-stake examinations, these factors need to be examined in a social-cultural environment that is distinct from other European and western learning environments. Despite previous research demonstrating that growth mindset, grit and FL enjoyment can influence language learning positively, these three factors are understudied in the field of FL learning, especially growth mindset and grit among Chinese EFL university students. Moreover, there is scant empirical evidence on the mechanism that underlies the links between these factors. Therefore, this study proposed a parallel mediation model to address the relationship among growth mindset, grit, FL enjoyment, and ELP among Chinese EFL university students from the perspective of positive psychology.

## Review of Literature

The following section will present a brief review of the literature on the variables that were explored in the study. They include factors such as growth mindset, grit, and FL enjoyment.

### Growth Mindset and ELP

Mindset refers to the set of beliefs that a person has about himself or herself, which is also known as implicit theories of intelligence (Dweck, 2006). Recently, several scholars have applied the theory of mindset to the field of FL learning, arguing that mindsets are domain-specific and language mindsets are different from mindsets in other domains (Mercer and Ryan, 2010; Ryan and Mercer, 2012; Lou and Noels, 2016; Khajavy et al., 2021a). The language mindset can be defined as language learners' beliefs about their language learning intelligence (Lou and Noels, 2017). Individuals who possess a fixed language mindset view language learning ability as innate, and therefore unable to be improved by effort. On the other hand, those who adhere to a growth language mindset hold the belief that a person's ability to learn languages can be enhanced through diligence and effort (Mercer and Ryan, 2010; Ryan and Mercer, 2012).

Growth mindset has been shown to influence students' academic performance positively in numerous studies. For example, two studies conducted by Romero et al. (2010) and Paunesku et al. (2011) indicated that growth mindset had a positive impact on students' performance in mathematics and reading tests. After the growth mindset intervention, students' scores in mathematics and reading tests improved. Likewise, Wang et al. (2020) revealed that growth mindset was positively correlated with Chinese adolescents' academic achievements. The study also showed the mediating effect of reasoning ability and the moderating effect of self-affirmation on the association between growth mindset and academic achievement.

In the field of language learning, growth mindset has been reported to be a significant predictor of ELP among Chinese high school students (Rui and Muthikrishnan, 2019; Wang et al., in press). In addition, research has revealed that growth L2 reading mindset significantly predicted L2 reading performance (Khajavy et al., 2021b). Khajavy et al. (2021a) also found that growth language mindset positively and significantly predicted L2 achievement whereas fixed language mindset did not significantly predict language achievement. Ryan and Mercer (2012) argued that language learners can potentially benefit tremendously from embracing the growth mindset because it has the potential to play a critical role in how learners approach language learning, what their goals are, and how they determine their success and level of achievement.

Despite the above favorable findings, other meta-analysis studies have also indicated that growth mindset has only a modest impact on students' academic performance (Burnette et al., 2013; Sisk et al., 2018). Lou and Noels (2019) argued that the association between mindsets and students' academic performance might be quite complicated as some studies have reported weak effects of mindsets on students' academic success. This study has however hypothesized that grit and FL enjoyment may play mediating roles in the relationship between growth mindset and ELP.

### The Mediating Role of Grit

Defined as “perseverance and passion for long-term goals” (Duckworth et al., 2007, p. 1087), grit is known as a non-cognitive personality trait that is critical to personal success (Duckworth et al., 2007; Duckworth and Quinn, 2009). Duckworth et al. (2007) stated that grit is composed of two underlying sub-constructs: perseverance of effort (POE) and consistency of interests (COI). POE refers to the sustained effort over time toward the long-term goals, whereas COI represents the enthusiasm for pursuing long-term goals. One of the features of gritty people is that they formulate long-term aspirations for themselves and remain loyal to them despite setbacks and challenges (Duckworth and Gross, 2014).

Previous studies have indicated that growth mindset and grit are positively associated with each other. For instance, Teimouri et al. (2020) reported that grit was positively correlated with growth mindset, and negatively correlated with fixed mindset. They argued that individuals with higher levels of grit tended to believe that they can become smarter through putting more effort. Khajavy et al. (2021a) revealed positive effects of language growth mindset on one component of grit (i.e., POE), but no direct effect on the other factor which was COI. Their study also showed that a fixed language mindset was a negative predictor of COI. They explained that the meaning of failure appears to be the link between grit and language mindset. For an individual with a fixed mindset, failure indicates a lack of intelligence or ability, and that a student lacks the necessary skills to be a successful language learner. For someone who possesses a growth mindset, on the other hand, failure is an essential component of language learning and serves as a potential chance to improve and progress (Khajavy et al., 2021a).

Previous studies on the relationship between grit and academic success have yielded mixed results. A number of studies indicated that grit was positively correlated with students' academic achievement (Duckworth and Quinn, 2009; Akos and Kretchmar, 2017). In the field of language learning, L2 grit has been found to be positively linked to language achievements (Teimouri et al., 2020). Sudina and Plonsky (2021) found that L2-specific POE significantly predicted language achievement. Lee (2020) showed that POE significantly predicted students' WTC. Wei et al. (2019) and Liu and Wang (2021) found a positive association between Chinese middle and high school students' grit and their FL performance. As discussed above, this study proposed that grit could be a mediating factor in how growth mindset predicts ELP.

However, Bazelais et al. (2016) and Usher et al. (2019) found no significant relationship between grit and academic achievement. In the field of language learning, Kramer et al. (2018) found none of the two sub-constructs of grit were linked to students' performance in the reading test. Khajavy et al. (2021a) reported non statistically significant associations between two sub-constructs of grit and L2 achievement. These inconclusive results on the association between grit and academic achievement has been a catalyst for researchers to investigate their relationship further. In this regard, this study aimed to examine if grit could mediate the relationship between Chinese EFL learners' growth mindset and their ELP.

### The Mediating Role of FL Enjoyment

According to Seligman and Csikszentmihalyi (2000), positive emotion is one of the foundations of positive psychology. Among the several positive emotions, one aspect that has recently come to the forefront in FL learning is FL enjoyment (MacIntyre et al., 2019; Dewaele and Li, 2020; Botes et al., in press). Dewaele and MacIntyre (2016) conceptualized enjoyment as a “complex emotion, capturing interacting dimensions of challenge and perceived ability that reflect the human drive for success in the face of difficult tasks” (p. 217). Fredrickson's (2001) broaden-and-build theory emphasizes the value of positive emotions for people's wellbeing and flourishing. According to the theory, positive emotions, such as enjoyment, interest and happiness can “broaden people's momentary thought-action repertoires and build their enduring personal resources, ranging from physical and intellectual resources to social and psychological resources” (Fredrickson, 2001, p. 219). MacIntyre and Gregersen (2012b) noted that enjoyment facilitates language learners in widening their thought-action repertoire, allowing them to absorb more information and building language resources. Moreover, the control-value theory also acknowledges that enjoyment as a form of positive emotions has a favorable impact on students' academic achievement (Pekrun et al., 2007). FL enjoyment promotes foreign language learning by encouraging students to be creative and experiment with a new language (Dewaele and MacIntyre, 2016).

In terms of the measurement of FL enjoyment, Dewaele and MacIntyre (2014) designed a 21-item Foreign Language Enjoyment Scale (FLES). In order to identify the applicability of FLES in the Chinese EFL context, Li et al. (2018) conducted the first thorough investigation of the FL enjoyment with Chinese EFL students and validated an 11-item Chinese Version of the Foreign Language Enjoyment Scale (CFLES). Similarly, Jin and Zhang (2018) also surveyed Chinese EFL students and established a 17-item English Classroom Enjoyment Scale (ECES) based on the original 21-item FLES. Subsequently, Jin and Zhang (2019) reduced the 17-item ECES to a 16-item ECES and compared their 16-item ECES to Li et al. (2018)'s 11-item CFLES, claiming that the former had “a more solid dimensional division and better psychometric properties” (p. 14). Based on this rationale and given the Chinese social-cultural learning environment, this study utilized Jin and Zhang (2019)'s 16-item measurement scale.

Although the relationship between growth mindset and FL enjoyment lacks empirical evidence, their relationship has been indicated in several studies on growth mindset and academic emotions. For example, Teimouri et al. (2020) noted that students with a growth mindset displayed less anxiety and more joy in language learning. In their study, growth mindset was reported to be negatively associated with language anxiety, but positively correlated with joy. Khajavy et al. (2021b) found that learners with a growth L2 reading mindset experienced more enjoyment in L2 reading comprehension, whereas students who possessed a fixed L2 reading mindset experienced less enjoyment. Likewise, Wang H. et al. (2021) reported that Chinese undergraduate students' growth language mindsets were closely connected to more enjoyment in English class while their fixed language mindsets were associated with more boredom in English class. The existing research also suggested that students who possess a growth mindset were more inclined to experience higher level of positive emotions in the learning process (Hsieh et al., 2012; Yeager and Dweck, 2012; Zeng et al., 2016). As outlined in the control-value theory of achievement emotions (Pekrun, 2006), positive emotions are experienced when one believes he or she has control over the outcomes (high control-related appraisal) and values the task (high value-related appraisal). Appraisals of low control and low value, by contrast, cause negative emotions (King et al., 2012). As language learners with a growth mindset view their language learning ability as controllable, and therefore able to be improved by hard work, they tend to have higher control-related appraisals. Therefore, compared with students with a fixed mindset, it is more likely for those with a growth mindset to experience more positive emotions.

It has repeatedly been demonstrated that the FL enjoyment is associated with language learners' academic performance. For example, Dewaele and Alfawzan (2018) examined the impact of FL enjoyment and FL classroom anxiety on students' FL performance. Their findings indicated a significant and positive link between learners' FL enjoyment and their test performance, which was slightly stronger than the negative link between students' FL classroom anxiety and their FL performance. Likewise, Li et al. (2020) investigated the interaction between Chinese EFL learners' FL enjoyment and FL classroom anxiety and reported a positive association between students' FL enjoyment and their self-perceived proficiency as well as a negative relationship between students' FL classroom anxiety and their self-perceived proficiency. Jin and Zhang (2018) and Guo (2021) also found that FL enjoyment was linked to Chinese EFL learners' academic success. More recently, Botes et al. (in press)'s meta-analysis revealed a moderate positive relationship between FL enjoyment and WTC, and FL enjoyment and academic performance in the FL. From the above discussion, this study proposed that FL enjoyment could act as a mediating variable between growth mindset and ELP.

### The Present Study

Even though there is a growing interest in the significant roles of growth mindset, grit, and FL enjoyment in language learning, to the best of our knowledge, no study has explored the simultaneous effects of these factors on language achievement. While studies have found that growth mindset can contribute to language learning, the predictive mechanisms for growth mindset in connection with ELP remain unknown. Previous research has insufficiently explored the possibility that grit and FL enjoyment could mediate the association between growth mindset and ELP.

To address the prior research limitations, this study aimed to examine the link between growth mindset and ELP of Chinese EFL university students with a parallel mediation model. It was assumed that growth mindset may relate to grit and FL enjoyment, which in turn associate with ELP. To this end, the following research questions were examined:

RQ1: What are the relationships between Chinese EFL university students' growth mindset, grit, FL enjoyment, and ELP?
RQ2: Do Chinese EFL university students' grit and FL enjoyment mediate the relationship between growth mindset and ELP?

## Methods

### Participants

A total of 388 EFL students were randomly selected from different faculties in one public university in Northern China. They were all non-English majors in their second year (sophomores). There were 183 female students and 205 male students, and their ages ranged from 17 to 21 (Mean = 19.88, SD = 0.703). The respondents were informed that the data would only be used for the research purpose. Keeping in line with ethics in research, prior permission was obtained from the university authorities to conduct the study and each respondent was also required to sign the consent form to participate in the research. They were also assured that no names would be required when responding to the questionnaire.

### Instruments

#### Language Mindsets Inventory

To assess participants' growth mindset, the Language Mindsets Inventory (LMI) (Lou and Noels, 2017) was employed. Lou and Noels (2017) designed an 18-item scale for measuring the growth and fixed mindset in language learning. However, the nine items measuring fixed mindset were not the concern of this study, therefore, only nine items were employed to measure students' growth mindset. The questionnaire consisted of three sub-constructs: general language intelligence beliefs (GL), second language aptitude beliefs (SL), and age sensitivity beliefs about language learning (AS). The participants were required to respond to all the items based on a five-point Likert-scale format ranging from 1 (strongly disagree) to 5 (strongly agree). Examples of items included the following: “*No matter who you are, you can significantly change your language intelligence level”* and “*Everyone could do well in foreign language if they try hard, whether they are young or old.”* In this present study, the Cronbach's alpha for growth mindset and its sub-constructs were 0.889, 0.885, 0.837, and 0.852, respectively.

#### L2 Grit Scale

The L2 Grit Scale (Teimouri et al., 2020) was utilized to assess participants' grit in English learning. The L2-specific grit scale was composed of two sub-constructs—POE and COI. Sample items included: “*Now that I have decided to learn English, nothing can prevent me from reaching this goal”* and “*I am a diligent English language learner.”* This study explored 8 items from the original nine-item scale. Here again participants responded to items based on a five-point Likert scale ranging from a score of 1 to 5 where a score of 1 indicated strongly disagreement whilst a score of 5 indicated strong agreement to the item. In this current study, the Cronbach's alpha for grit and its sub-constructs were 0.886, 0.879, and 0.831, respectively.

#### English Classroom Enjoyment Scale

Participants' enjoyment in learning English was assessed by the ECES (Jin and Zhang, 2019), which was adapted from Dewaele and MacIntyre (2014)'s 21-item FLES. The ECES was composed of three sub-constructs, namely, enjoyment of teacher support (TS), enjoyment of student support (SS), and enjoyment of English Language Learning (EL). It has been verified with reliability and validity in the Chinese context and used in relevant research (Jin and Zhang, 2019). There were 15 items with a five-point Likert-scale ranging from 1 (strongly disagree) to 5 (strongly agree). Example of the items were as follows: “*The teacher is supportive”* and “*In class, I feel proud of my accomplishments.”* In the present study, the Cronbach's alpha for FL enjoyment and its sub-constructs were 0.919, 0.821, 0.907, and 0.846, respectively.

#### College English Test-Band 4

Participants' English language performance (ELP) was measured based on their CET-4 scores. The CET-4 is known as the most influential and highly participated English language test conducted in all China's institutions of higher education (Ma, 2014). All non-English majors are required to take CET-4. With a total score ranging from 220 to 710, the CET-4 test comprises four sections: writing (15%), translation (15%), listening comprehension (35%), and reading comprehension (35%). CET-4 has been reported to have high reliability and validity (Zhang and Chen, 2015).

### Data Collection Procedures

The ethical approval for this research was obtained from the Ethical Committee of the Faculty of Education, Languages and Psychology, SEGi University. The permission was also obtained from the university. The participants were assured that the data would be utilized for the research purpose only and they were required to sign the consent form. Next, questionnaires were distributed to 388 participants through the online questionnaire platform Wenjuanxing. Participants completed the online survey in ≈10–15 min. They were allowed to ask the researchers through email if they had questions about the questionnaires for better understanding.

### Data Analysis

Data cleaning including checking missing values, identifying outliers, and normality tests were conducted using SPSS 26.0. Confirmatory Factor Analysis (CFA) was conducted to verify the validity and reliability of this study's measurement model using AMOS 24.0. Next, structural equation modeling (SEM) was employed for path analysis. Lastly, Bias-corrected bootstrap tests with 95% confidence intervals were performed to examine the mediation effects.

## Results

Before conducting the main analyses, data cleaning was performed. It was found that there were no missing values in the dataset. Next, the outliers of 388 dataset were assessed using boxplots. No cases were found to have extreme outliers. The final sample of 388 was used for further analysis.

### Confirmative Factor Analysis

Initially, a CFA was performed to establish the construct validity. There were three second-order constructs in the measurement model. GL, SL, and AS were the three sub-constructs that assessed the growth mindset construct. Two sub-constructs determined the grit construct: POE and COI. In addition, TS, EL, and SS were the sub-constructs that evaluated the FL enjoyment construct. Table 1 summarizes the results of the initial CFA model.

**Table 1:** Unstandardized and standardized estimates of the initial CFA model.

	**Unstandardized Estimate**				**Standardized**
	**Estimate**	**S.E**.	**C.R**.	***P***	**Estimate**
FLE4 ← EL	1.000				0.819
FLE5 ← EL	1.082	0.060	17.890	0.000	0.797
FLE6 ← EL	0.959	0.057	16.869	0.000	0.764
FLE7 ← EL	0.962	0.056	17.189	0.000	0.775
FLE8 ← EL	0.317	0.069	4.624	0.000	0.242
FLE9 ← EL	1.040	0.060	17.196	0.000	0.775
FLE10 ← EL	1.042	0.058	17.865	0.000	0.797
FLE11 ← EL	0.439	0.069	6.392	0.000	0.330
FLE3 ← TS	0.795	0.057	13.957	0.000	0.693
FLE2 ← TS	0.911	0.056	16.165	0.000	0.798
FLE1 ← TS	1.000				0.845
FLE14 ← SS	0.888	0.055	16.242	0.000	0.754
FLE13 ← SS	0.667	0.043	15.438	0.000	0.724
FLE12 ← SS	1.000				0.854
FLE15 ← SS	0.863	0.055	15.714	0.000	0.734
GM6 ← SL	1.052	0.067	15.778	0.000	0.807
GM5 ← SL	0.916	0.060	15.367	0.000	0.783
GM4 ← SL	1.000				0.796
GM9 ← AS	1.087	0.064	17.071	0.000	0.826
GM8 ← AS	1.065	0.065	16.277	0.000	0.785
GM7 ← AS	1.000				0.829
GM1 ← GL	1.000				0.925
GM2 ← GL	0.902	0.041	21.786	0.000	0.835
GM3 ← GL	0.912	0.046	19.966	0.000	0.792
GR1 ← POE	1.000				0.771
GR2 ← POE	1.276	0.077	16.496	0.000	0.815
GR3 ← POE	0.349	0.081	4.303	0.000	0.230
GR4 ← POE	1.269	0.078	16.266	0.000	0.805
GR5 ← POE	1.298	0.077	16.862	0.000	0.832
GR6 ← COI	1.000				0.813
GR7 ← COI	1.058	0.069	15.248	0.000	0.776
GR8 ← COI	1.020	0.067	15.275	0.000	0.777

**Figure 1:**
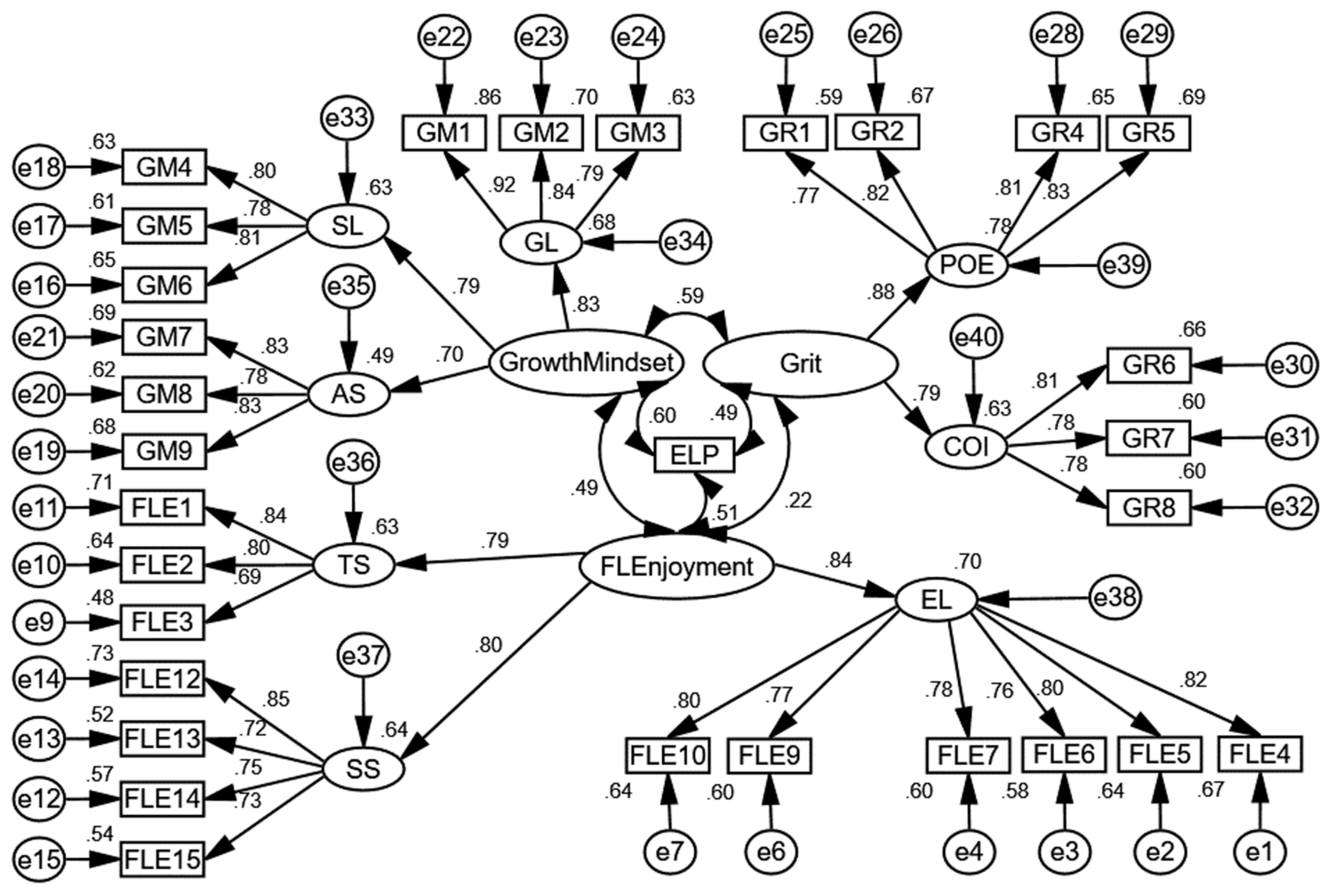
The final CFA model with standardized estimates.

As shown in Table 1, all factor loadings in the CFA model were significant. However, the items labeled FLE8 and FLE11 from FL enjoyment as well as GR3 from grit had factor loadings of 0.242, 0.330, and 0.230, respectively. These three items were removed from the model as their factor loadings were lower than 0.5. Figure 1 shows the final CFA model.

To gauge the model's fit, several indicators of goodness-of-fit were examined. Table 2 presents the measures of model fit and the threshold level suggested by Hu and Bentler (1999).

**Table 2:** Evaluation of the CFA model fit.

**Measure**	**Estimate**	**Threshold**	**Evaluation**
CMIN	422.398	–	–
DF	392.000	–	–
CMIN/DF	1.078	Between 1 and 3	Excellent
CFI	0.995	>0.95	Excellent
TLI	0.995	>0.95	Excellent
SRMR	0.035	<0.08	Excellent
RMSEA	0.014	<0.06	Excellent

As shown in Table 2, all the fitness indexes for the CFA model achieved the excellent level. Next, the reliability and validity of the CFA model were assessed (Table 3).

**Table 3:** Reliability and validity of the CFA model.

			**Fornell-Larcker Criterion**			
	**CR**	**AVE**	**FL Enjoyment**	**Growth Mindset**	**Grit**	**ELP**
FL Enjoyment	0.851	0.656	**0.810**			
Growth Mindset	0.818	0.601	0.487^***^	**0.776**		
Grit	0.825	0.703	0.215^**^	0.591^***^	**0.838**	
ELP	–	–	0.514^***^	0.602^***^	0.486^***^	–

** *Correlation is significant at p < 0.01*.

*** *Correlation is significant at p < 0.001*.

*The bold values means the square root of AVE for each construct*.

As indicated in Table 3, the composite reliability (CR) values and average variance explained (AVE) for each construct exceeded the threshold values of 0.7 and 0.5, respectively. Accordingly, the reliability and convergent validity for the final CFA model were achieved. Moreover, the discriminant validity for the final CFA model was established as the square root of AVE (the bold values in the table) for each construct was greater than the correlations between the respective constructs (Fornell and Larcker, 1981).

Upon examination of the correlations (values not in bold under Fornell-Larcker Criterion), significant relationships were found among all constructs. Growth mindset was reported to be strongly associated with grit (*r* = 0.591, *p* < 0.001), moderately related to FL enjoyment (*r* = 0.487, *p* < 0.001), and strongly associated with ELP (*r* = 0.602, *p* < 0.001). Moderate correlations were found between grit and ELP (*r* = 0.486, *p* < 0.001), as well as FL enjoyment and ELP (*r* = 0.514, *p* < 0.001).

Next, the data from the final CFA model were imputed. The descriptive statistics for the imputed constructs are summarized in Table 4.

**Table 4:** Descriptive statistics for the imputed constructs.

	***N***	**Minimum**	**Maximum**	**Mean**	**SD**	**Skewness**	**Kurtosis**
COI	388	1.04	4.57	3.3223	0.83823	−0.702	−0.145
POE	388	0.97	4.10	2.9876	0.78356	−1.108	0.473
Grit	388	1.18	4.08	3.0432	0.65031	−1.018	0.540
GL	388	1.26	5.43	4.0076	1.09344	−1.066	0.391
AS	388	1.20	4.50	3.3690	0.76256	−0.504	−0.370
SL	388	1.31	4.96	3.6366	0.86231	−0.450	−0.367
Growth Mindset	388	1.95	6.13	4.6352	0.85108	−0.758	0.511
SS	388	1.28	5.49	4.1133	0.99007	−1.047	0.403
TS	388	1.16	5.12	3.7595	0.81196	−1.071	1.148
EL	388	1.21	4.93	3.5425	0.85384	−0.606	−0.251
FL Enjoyment	388	1.32	4.61	3.3449	0.62966	−0.913	0.880
ELP	388	307	593	419.46	51.541	0.499	0.641

As shown in Table 4, the values of skewness and kurtosis were all within the range of the absolute value of 2, indicating that the imputed data were normally distributed.

### Structural Equation Modeling

**Figure 2:**
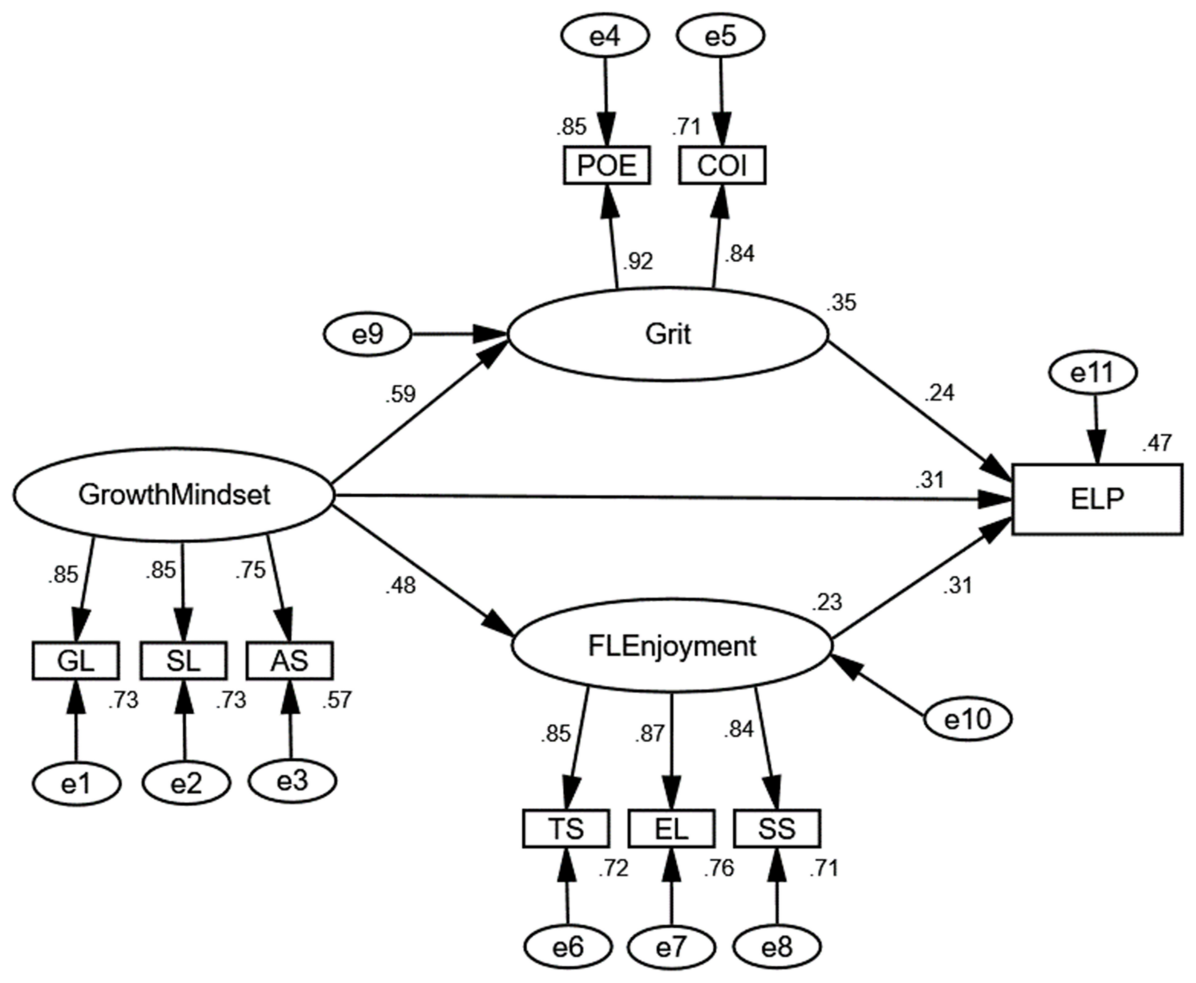
The structural model with standardized estimates.

Following the validation of the CFA model, SEM was performed to test the direct effects and indirect effects between growth mindset, grit, FL enjoyment, and ELP. The structural model with standardized estimates is presented in Figure 2.

As reported in Figure 2, all the factor loadings in the structural model exceeded the value of 0.5. In addition, the model fit for the structural model met the excellent level (Chisq/df = 1.069 < 3.0, RMSEA = 0.013 < 0.06, SRMR = 0.029 < 0.08, CFI = 0.999 > 0.95, TLI = 0.999 > 0.95). The results of the path analysis for the structural model are presented in Table 5.

**Table 5:** Path coefficients of the structural model.

	**Weight**	**S.E**.	**C.R**.	***P***	**β**
Grit ← Growth Mindset	0.457	0.042	10.94	0.000	0.590
FL Enjoyment ← Growth Mindset	0.358	0.041	8.626	0.000	0.485
ELP ← Growth Mindset	17.028	3.405	5.000	0.000	0.307
ELP ← Grit	17.017	3.914	4.348	0.000	0.238
ELP ← FL Enjoyment	23.429	3.674	6.377	0.000	0.312

As reported in Table 5, all five paths in the structural model were significant at the 0.001 level. The path from growth mindset to grit (β = 0.590, *p* < 0.001), to FL enjoyment (β = 0.485, *p* < 0.001), and to ELP (β = 0.307, *p* < 0.001), the path from grit to ELP (β = 0.238, *p* < 0.001), and the path from FL enjoyment to ELP (β = 0.312, *p* < 0.001), were all significant.

### Mediation Analysis

Bias-corrected bootstrap tests with 95% confidence intervals were performed to determine the mediating effects of grit and FL enjoyment on the relationship between growth mindset and ELP. Gender and age were included as control variables. Table 6 summarizes the bootstrapping analysis results.

**Table 6:** Bootstrapping analysis of the mediating effects (controlling for gender and age).

	**Unstandardized Estimate**	**Boot SE**	**Bias-Corrected 95%CI**		**Standardized Estimate**
			**Lower**	**Upper**	
Total effects	33.203	3.208	28.185	38.761	0.599^***^
Direct effects	17.086	3.740	10.881	23.398	0.308^***^
Indirect effects	16.117	2.396	12.405	20.206	0.291^***^
Grit as the mediator	7.739	2.003	4.326	11.002	0.140^**^
FL Enjoyment as the mediator	8.378	1.486	6.219	11.167	0.151^***^

** *p < 0.01*.

*** *p < 0.001*.

As displayed in Table 6, there was no zero included in the 95% confidence intervals. The indirect effects of growth mindset on ELP through grit (0.140, *p* < 0.01, 95% CI: 4.326–11.002) and FL Enjoyment (0.151, *p* < 0.001, 95% CI: 6.219–11.167) were significant, while the direct effects (0.308, *p* < 0.001, 95% CI: 10.881–23.398) and total effects (0.599, *p* < 0.001, 95% CI: 28.185–38.761) of growth mindset on ELP were also significant. Therefore, these findings suggested that grit and FL enjoyment partially mediated the relationship between growth mindset and ELP. Furthermore, the indirect effects took up 48.6% (0.291/0.599) of the total effects of growth mindset on ELP.

## Discussion

### Correlations Between Growth Mindset, Grit, FL Enjoyment, and ELP

The first research question of the present study sought to examine the relationships between growth mindset, grit, FL enjoyment, and ELP. The results showed that these constructs were significantly and positively correlated with each other.

To begin with, growth mindset was found to be positively associated with grit, which echoed the results of existing research (Teimouri et al., 2020; Khajavy et al., 2021a). These findings suggested that when language learners possessed a growth mindset, they were more likely to put more effort in learning English. Khajavy et al. (2021a) further explained that when one holds a growth mindset perspective on language learning, they study harder and are more resilient to difficulties in order to enhance their language skills.

Moreover, a significant relationship between growth mindset and FL enjoyment was found, meaning that students with a growth mindset were more inclined to enjoy English learning. Similar findings in the field of language learning were also reported by Khajavy et al. (2021b); Wang H. et al. (2021), and Teimouri et al. (2020). The findings of this study also concurred with the previous research indicating that growth mindset was related to positive emotions among students (Hsieh et al., 2012; Yeager and Dweck, 2012; Zeng et al., 2016). In light of the control-value theory of achievement emotions (Pekrun, 2006), individuals who are positive about their language learning ability view their language skills as something under their control. Consequently, they might have more positive emotions as they hold the positive belief that they can improve their skills and achieve their goals by putting enough effort.

Additionally, the results of the present study revealed a positive correlation between growth mindset and ELP, which illustrated that students who were positive about their language learning ability were more inclined to experience higher language achievements. In the field of language learning, the result of this study corroborated the findings from other studies (Rui and Muthikrishnan, 2019; Hassanzadeh et al., 2020; Khajavy et al., 2021a,b; Wang et al., in press) exhibiting that growth mindset significantly predicted language achievement. This might be because individuals who believe that capability can be strengthened *via* work and dedication are more inclined to put out effort, seek challenges, and persevere through failures, resulting in greater language accomplishment (Lou and Noels, 2016; Bai et al., 2020). Moreover, this result was also aligned with the existing research findings that recorded the positive impact of growth mindset on students' academic achievements (Tarbetsky et al., 2016; Mouratidis et al., 2017; Sisk et al., 2018; Wang et al., 2020).

Besides, grit was found to positively correlate with ELP, suggesting that students who were grittier in learning English were prone to gaining higher scores in English tests. Similar to this present study, Wei et al. (2019) and Liu and Wang (2021) found a positive association between Chinese middle and high school students' grit and their FL performance. Similar results were also reported by Lee (2020) that grit (the POE sub-construct) predicted students' WTC as well as research in other EFL contexts (Sudina et al., 2020; Teimouri et al., 2020). Those possessing a higher level of grit are believed to focus better and are less deterred by mistakes and losses, thus they can optimize their potential and ability (Credé et al., 2017). Additionally, previous research also confirmed the positive role of grit in students' academic achievements (Perkins-Gough, 2013; Macnamara et al., 2014; Aparicio et al., 2017; Tang et al., 2019).

Finally, FL enjoyment was found to be positively associated with ELP, which implied that learners who enjoyed learning English would be able to achieve higher learning gains. This result was in line with the prior research reporting that there existed a positive correlation between FL enjoyment and FL achievements among Chinese middle school students (Wei et al., 2019), high school students (Jin and Zhang, 2018; Li, 2019; Liu and Wang, 2021), and university students (Zhang et al., 2020; Guo, 2021), Korean students (Lee, 2020), in other international contexts (Dewaele and MacIntyre, 2014; Dewaele and Alfawzan, 2018), and in the recent meta-analysis (Botes et al., in press). Based on the broaden-and-build theory (Fredrickson, 2001), when students enjoy learning a foreign language, their horizons of thought are expanded, and they tend to apply various learning strategies, which promotes language learning efficiency (Jin and Zhang, 2018). As a result, individuals who experience more enjoyment in learning English are more inclined to become successful language learners (Li, 2019).

In summary, given the findings above, this study found that there were significant relationships between growth mindset and the two other positive factors (grit and FL enjoyment), and between the three positive factors and ELP. Hence the study revealed that the association between growth mindset and ELP was partially mediated by grit and FL enjoyment. That aspect will be discussed in the section below.

### Grit and FL Enjoyment as Mediators

The second research question of this present study aimed to determine the mediating effects of grit and FL enjoyment on the association between growth mindset and ELP. The mediation analysis showed that after controlling for gender and age, grit and FL Enjoyment played partial mediating roles, indicating that growth mindset not only directly predicted ELP, but also indirectly influenced ELP through the other two positive factors (i.e., grit and FL Enjoyment).

It was found that grit mediated the link between growth mindset and ELP, indicating that students who held the belief that their English language ability could be improved were more inclined to possess a higher level of grit and perseverance in learning English, which in turn contributed to higher language achievements. Individuals with a growth mindset are prone to blaming their failure on their personal factors such as lack of hard work, as a result of which, they are likely to be motivated to achieve their goals by putting in more effort (Rattan et al., 2015). Therefore, growth mindset can improve ELP by increasing the degree of grit. This finding further highlighted the significant role of grit in obtaining higher language achievements. In the process of language learning, language learners with more grit often persevere in facing obstacles, will put in more effort to improve their English language skills, and will finally obtain better language performance (Lan and Moscardino, 2019; Wei et al., 2019).

Similarly, this study also revealed that growth mindset had indirect effects on ELP via FL enjoyment, suggesting that students with a growth mindset tended to find learning English enjoyable, which consequently could lead to enhanced language proficiency. If students possess confidence in their language learning ability, they view failure as evidence that they are not putting in enough time and effort in learning, as a result of which, they will put more effort to achieve their goals even when facing challenges (Bai and Wang, 2020). For language learners with a growth mindset, making sufficient effort in language learning usually leads to positive evaluation of their own behaviors, causing FL enjoyment and other positive feelings (Wei et al., 2019). Consequently, when students possess a growth mindset, they tend to experience a higher level of FL enjoyment. Enjoyment is a positive emotion that arises from overcoming one's constraints and extending beyond oneself in order to achieve something challenging (Dewaele and MacIntyre, 2016). Based on the broaden-and-build theory (Fredrickson, 2001) which postulates that positive emotions not only broaden one's awareness but also encourage one to cope with negative emotions, FL enjoyment can help individuals gain broader perspectives and increase their capacity to absorb new material in language learning. This is also conducive to boosting language learners' resilience and self-esteem (MacIntyre and Gregersen, 2012a). Additionally, the positive impact of FL enjoyment on English language performance is also supported by the control-value theory (Pekrun, 2006) which hypothesizes that learners' level of perceived control over learning achievement via strategies such as motivation to learn, learning strategies and self-regulatory strategies can enhance academic achievements. Students can also benefit from FL enjoyment by gaining positive strength, reducing stress, and increasing their motivation and enthusiasm in English learning (Piniel and Albert, 2018). Moreover, the mediating role of FL enjoyment in the association between growth mindset and ELP found in this study echoed the existing research reporting the mediating effects of FL enjoyment on the association between trait emotional intelligence (Li, 2019), motivation (Zhang et al., 2020), grit (Wei et al., 2019; Liu and Wang, 2021) and language learners' academic performance, respectively.

## Conclusion and Implications

This study attempted to obtain a deeper understanding of the potential relationship between growth mindset and ELP with the focus on the roles of grit and FL enjoyment as the mediating variables among Chinese EFL university students. Findings of the study demonstrated that the association between growth mindset and ELP was partially mediated by grit and FL enjoyment, implying that higher levels of growth mindset contributed to greater ELP *via* the increase in the levels of grit and FL enjoyment.

At this point, it is pertinent to note that these findings are not without its share of limitations. The first limitation is the scope of this study. It cannot be denied that there are various positive psychological and emotional factors that can affect language learning. This study only focused on three factors, namely growth mindset, grit, and FL enjoyment. It would be beneficial for future research to explore other potential factors that have not examined in this study. Another limitation lies in the cross-sectional and correlational design that was adopted in this study, as a result of which, causal relationships among variables could not be detected. It is suggested that experimental and longitudinal designs be utilized in future studies to examine the role of growth mindset, grit, and FL enjoyment in students' language learning achievements.

Nevertheless, despite some limitations, this study is ecologically valid as the findings of the study can be generalized to some language learning scenarios, especially in stress-fueled exam-oriented learning environments in Asia-Pacific regions, like China where high achievement is considered a student's obligation to family reputation and personal value and self-worth. With positive factors such as growth mindset, grit, and FL enjoyment influencing ELP, the findings imply that it is perhaps crucial that language teachers, educational material developers, and school administrators realign language policies, curricula, and assessments that postulate a more balanced and holistic education. Such a move will embrace learners' wellbeing and foster learners' positive beliefs (e.g., growth mindset), positive personality traits (e.g., grit), and positive emotions (e.g., FL enjoyment) despite the competitive social-cultural learning environment. Once a balance is maintained between the two (i.e., assessment of learning and assessment for learning), students may be able to address the challenges in learning an L2 or FL. Given below are the implications provided by this study.

The study reported a positive direct effect of growth mindset on ELP, and an indirect effect of growth mindset on ELP through the two parallel mediators of grit and FL enjoyment. In this case, growth mindset appears to be a primary factor in improving students' ELP. In order to achieve better results in FL learning, students need to embrace personal wellbeing and holistic learning despite learning within a challenging exam-oriented context. Therefore, it is important that growth mindset should be instilled in students given its essential role in facilitating language learning. To begin with, it is the responsibility of the teachers to cultivate students' ability to embrace negative comments. It is critical that language learners learn how to treat negative feedback and utilize it as a springboard for making progress in language learning. Students should see modest amounts of criticism from suitable sources as positive and constructive. Some language learners are overly sensitive and lack the necessary perspective to accept constructive criticism. However, teachers teaching in competitive stressful learning environments, are advised to balance assessment “of” and “for” learning via both effective formative and summative assessment frameworks. In such holistic learning environments, teachers can provide more constructive feedback with care and kindness, and emphasize the specific measures students should take to achieve the desired outcome. Furthermore, it is critical that teachers emphasize the constructive function of failures instead of rejecting them as an indication of incompetence. Teachers should encourage their students to embrace all feedback positively and learn from their mistakes by doing the necessary corrections so that they can continue to enhance their language performance. More importantly, more attention should be paid to hard work rather than intelligence or ability (Khajavy et al., 2021a). Thus, when students complete a language task, teachers need to be generous and credit students for their effort and not ability.

With regards to the mediating effect of grit on the link between growth mindset and ELP that was reported by this study, it is believed that students should devote their effort and passion into language learning, especially those in the exam-oriented environment. There are several techniques and strategies for teachers to apply in the classroom to encourage grit in students. To start with, grit can be introduced by teachers into the language classroom to maximize language usage and ultimately improve learning outcomes. Lectures emphasizing the significance of “hard work” in the language learning process can be given to students. Meanwhile, teachers should offer encouragement and recognition to students who show perseverance in completing difficult tasks. Finally, teachers can share stories of language learners who excel in language learning owing to their dedication to learning a foreign language (Khajavy et al., 2021b). As a result, students will be inspired to study harder.

FL enjoyment was found to play a mediating role in the effect of growth mindset on ELP. It is hence suggested that more attention be paid to students' wellbeing and emotional state as EFL students in the exam-oriented environment have been found to experience negative emotions such as nervousness, pressure, and anxiety (Xiao and Carless, 2013; Dawadi, 2022). Firstly, teachers are encouraged to create a peaceful, nurturing, and uplifting environment by providing appropriate collaborative classroom activities where the interaction among students could be boosted, such as group discussion and role-playing. Likewise, there should be no “put-downs,” and learners who are willing to try should be praised for their courage to take risks in language learning. Secondly, in today's technology-driven world, educational material developers should endeavor to incorporate audio and video materials into classroom syllabus (Peng, 2019) and design some interactive and authentic language learning tasks that the students find enjoyable, which will aid teachers in creating a positive and enjoyable learning environment for students. Finally, the school administrators should de-emphasize the exam-orientated environment and allow teachers to be autonomous in assessing and evaluating language learning (Yang, 2021). As mentioned above, this can be conducted through a creative assessment framework like continuous formative assessment which postulates a less aggressive and stressful learning environment, leading to less anxiety among EFL students which can undermine their enjoyment in language learning.

In summary, the findings of this study have highlighted the significance of understanding how positive psychological and emotional variables may function to enhance students' language performance. It is perhaps timely for language teachers to stand up and address these variables so that they can alleviate FL anxiety and create stress-free, healthy, and positive learning environments in their EFL classrooms.

## Data Availability Statement

The raw data supporting the conclusions of this article will be made available by the authors, without undue reservation.

## Ethics Statement

The studies involving human participants were reviewed and approved by the Ethical Committee of the Faculty of Education, Languages and Psychology, SEGi University. The patients/participants provided their written informed consent to participate in this study.

## Author Contributions

XH conceived the study, analyzed the data, and drafted the manuscript. GS guided, reviewed, and revised the manuscript. XL helped collect and analyze the data. All authors contributed to the article and approved the submitted version.

## Conflict of Interest

The authors declare that the research was conducted in the absence of any commercial or financial relationships that could be construed as a potential conflict of interest.

## Publisher's Note

All claims expressed in this article are solely those of the authors and do not necessarily represent those of their affiliated organizations, or those of the publisher, the editors and the reviewers. Any product that may be evaluated in this article, or claim that may be made by its manufacturer, is not guaranteed or endorsed by the publisher.

## References

[B1] Akos P. Kretchmar J. (2017). Investigating grit at a non-cognitive predictor of college success. Rev. High. Educ. 40, 163–186. 10.1353/rhe.2017.0000

[B2] Aparicio M. Bacao F. Oliveira T. (2017). Grit in the path to e-learning success. Comput. Hum. Behav. 66, 388–399. 10.1016/j.chb.2016.10.009

[B3] Bai B. Wang J. (2020). The role of growth mindset, self-efficacy and intrinsic value in self-regulated learning and English language learning achievements. Lang. Teach. Res. 1–22. 10.1177/1362168820933190

[B4] Bai B. Wang J. Nie Y. (2020). Self-efficacy, task values and growth mindset: what has the most predictive power for primary school students' self-regulated learning in English writing and writing competence in an Asian Confucian cultural context? Camb. J. Educ. 51, 65–84. 10.1080/0305764X.2020.1778639

[B5] Bazelais P. Lemay D. J. Doleck T. (2016). How does grit impact college students' academic achievement in science? Eur. J. Sci. Math. Educ. 4, 33–43. 10.30935/scimath/9451

[B6] Botes E. Dewaele J. Greiff S. (in press). Taking stock: a meta-analysis of the effects of foreign language enjoyment. Stud. Second Lang. Learn. Teach.

[B7] Burnette J. L. O'boyle E. H. VanEpps E. M. Pollack J. M. Finkel E. (2013). Mind-sets matter: a meta-analytic review of implicit theories and self-regulation. Psychol. Bull. 139, 655. 10.1037/a0029531 22866678

[B8] Credé M. Tynan M. C. Harms P. D. (2017). Much ado about grit: a meta-analytic synthesis of the grit literature. J. Pers. Soc. Psychol. 113, 492. 10.1037/pspp0000102 27845531

[B9] Csikszentmihalyi M. (2008). Flow: The Psychology of Optimal Experience. ser. PS Series. New York, NY: HarperCollins.

[B10] Dawadi S. (2022). High-stakes test pressure and anxiety in the Nepalese English as a Foreign Language (EFL) learners. J. NELTA 26, 20–36. 10.3126/nelta.v26i1-2.45379

[B11] Dewaele J.-M. Alfawzan M. (2018). Does the effect of enjoyment outweigh that of anxiety in foreign language performance? Stud. Second Lang. Learn. Teach. 8, 21–45. 10.14746/ssllt.2018.8.1.2

[B12] Dewaele J.-M. Chen X. Padilla A. M. Lake J. (2019). The flowering of positive psychology in foreign/second language teaching and acquisition research. Front. Psychol. 10, 2128. 10.3389/fpsyg.2019.02128 31607981 PMC6769100

[B13] Dewaele J.-M. Li C. (2020). Emotions in second language acquisition: a critical review and research agenda. Foreign Lang. World 196, 34–49.

[B14] Dewaele J.-M. MacIntyre P. D. (2014). The two faces of Janus? Anxiety and enjoyment in the foreign language classroom. Stud. Second Lang. Learn. Teach. 4, 237–274. 10.14746/ssllt.2014.4.2.5

[B15] Dewaele J.-M. Witney J. Saito K. Dewaele L. (2018). Foreign language enjoyment and anxiety: the effect of teacher and learner variables. Lang. Teach. Res. 22, 676–697. 10.1177/1362168817692161

[B16] Dewaele J. -M. MacIntyre P. D. (2016). Foreign language enjoyment and foreign language classroom anxiety: The right and left feet of the language learner, in Positive psychology in SLA, eds Gregersen T. MacIntyre P. D. Mercer S. (Bristol: Multilingual Matters), 215–236. 10.21832/9781783095360-010

[B17] Duckworth A. Gross J. J. (2014). Self-control and grit: related but separable determinants of success. Curr. Dir. Psychol. Sci. 23, 319–325. 10.1177/0963721414541462 26855479 PMC4737958

[B18] Duckworth A. L. Peterson C. Matthews M. D. Kelly D. R. (2007). Grit: perseverance and passion for long-term goals. J. Pers. Soc. Psychol. 92, 1087. 10.1037/0022-3514.92.6.1087 17547490

[B19] Duckworth A. L. Quinn P. D. (2009). Development and validation of the Short Grit Scale (GRIT–S). J. Pers. Assess. 91, 166–174. 10.1080/00223890802634290 19205937

[B20] Dweck C. S. (2006). Mindset: How We Can Learn to Fulfill Our Potential. New York: NY: Random.

[B21] Fornell C. Larcker D. F. (1981). Evaluating structural equation models with unobservable variables and measurement error. J. Market. Res. 18, 39–50. 10.1177/002224378101800104

[B22] Fredrickson B. L. (2001). The role of positive emotions in positive psychology: the broaden-and-build theory of positive emotions. Am. Psychol. 56, 218. 10.1037/0003-066X.56.3.218 11315248 PMC3122271

[B23] Guo X. Díaz A. Liyanage I. (2016). Exploring the professional agency chasm in exam-driven English language education contexts, in Global Language policies and Local Educational Practices and Cultures, eds. O'Neill S. van Rensburg H. (Blue Mounds, WI: Deep University Press), 214–230.

[B24] Guo Y. (2021). Exploring the dynamic interplay between foreign language enjoyment and learner engagement with regard to EFL achievement and absenteeism: a sequential mixed methods study. Front. Psychol. 12, 766058. 10.3389/fpsyg.2021.766058 34721246 PMC8554116

[B25] Hassanzadeh L. Ahangari S. Tamjid N. H. (2020). The relationship between EFL learners' language mindsets and English achievement: engagement and self-regulation as mediators. Iran. J. Appl. Linguist. 23, 1–28.

[B26] Hsieh P.-H. Sullivan J. R. Sass D. A. Guerra N. S. (2012). Undergraduate engineering students' beliefs, coping strategies, and academic performance: an evaluation of theoretical models. J. Exp. Educ. 80, 196–218. 10.1080/00220973.2011.596853

[B27] Hu L.-T. Bentler P. M. (1999). Cutoff criteria for fit indexes in covariance structure analysis: conventional criteria versus new alternatives. Struct. Equ. Modeling: Multidiscip. J. 6, 1–55. 10.1080/10705519909540118

[B28] Jin Y. Zhang L. J. (2018). The dimensions of foreign language classroom enjoyment and their effect on foreign language achievement. Int. J. Biling. Educ. Biling. 24, 1–15. 10.1080/13670050.2018.1526253

[B29] Jin Y. Zhang L. J. (2019). A comparative study of two scales for foreign language classroom enjoyment. Percept. Mot. Skills 126, 1024–1041. 10.1177/0031512519864471 31345113

[B30] Khajavy G. H. MacIntyre P. D. Barabadi E. (2018). Role of the emotions and classroom environment in willingness to communicate: applying doubly latent multilevel analysis in second language acquisition research. Stud. Second Lang. Acquis. 40, 605–624. 10.1017/S0272263117000304

[B31] Khajavy G. H. MacIntyre P. D. Hariri J. (2021a). A closer look at grit and language mindset as predictors of foreign language achievement. Stud. Second Lang. Acquis. 43, 379–402. 10.1017/S0272263120000480

[B32] Khajavy G. H. Pourtahmasb F. Li C. (2021b). Examining the domain-specificity of language mindset: a case of L2 reading comprehension. Innov. Lang. Learn. Teach. 16, 1–13. 10.1080/17501229.2021.1956936

[B33] King R. B. McInerney D. M. Watkins D. A. (2012). How you think about your intelligence determines how you feel in school: the role of theories of intelligence on academic emotions. Learn. Individ. Differ. 22, 814–819. 10.1016/j.lindif.2012.04.005

[B34] Kramer B. Mclean S. Martin E. S. (2018). Student Grittiness: A Pilot Study Investigating Scholarly Persistence in EFL Classrooms. Osaka: Osaka Jogakuin College.

[B35] Lan X. Moscardino U. (2019). Direct and interactive effects of perceived teacher-student relationship and grit on student wellbeing among stay-behind early adolescents in urban China. Learn. Individ. Differ. 69, 129–137. 10.1016/j.lindif.2018.12.003

[B36] Lee J. Zhou M. (2015). The Asian American Achievement Paradox. New York, NY: Russell Sage Foundation.

[B37] Lee J. S. (2020). The role of grit and classroom enjoyment in EFL learners' willingness to communicate. J. Multiling. Multicult. Dev. 1–17. 10.1080/01434632.2020.1746319

[B38] Li C. (2019). A positive psychology perspective on Chinese EFL students' trait emotional intelligence, foreign language enjoyment and EFL learning achievement. J. Multiling. Multicult. Dev. 41, 246–263. 10.1080/01434632.2019.1614187

[B39] Li C. Dewaele J.-M. Jiang G. (2020). The complex relationship between classroom emotions and EFL achievement in China. Appl. Linguist. Rev. 11, 485–510. 10.1515/applirev-2018-0043

[B40] Li C. Jiang G. Dewaele J.-M. (2018). Understanding Chinese high school students' foreign language enjoyment: validation of the Chinese version of the foreign language enjoyment scale. System 76, 183–196. 10.1016/j.system.2018.06.004

[B41] Liu E. Wang J. (2021). Examining the relationship between grit and foreign language performance: enjoyment and anxiety as mediators. Front. Psychol. 12, 666892. 10.3389/fpsyg.2021.666892 34248761 PMC8264132

[B42] Lou N. M. Noels K. A. (2016). Changing language mindsets: implications for goal orientations and responses to failure in and outside the second language classroom. Contemp. Educ. Psychol. 46, 22–33. 10.1016/j.cedpsych.2016.03.004

[B43] Lou N. M. Noels K. A. (2017). Measuring language mindsets and modeling their relations with goal orientations and emotional and behavioral responses in failure situations. Mod. Lang. J. 101, 214–243. 10.1111/modl.12380

[B44] Lou N. M. Noels K. A. (2019). Promoting growth in foreign and second language education: a research agenda for mindsets in language learning and teaching. System 86, 102126. 10.1016/j.system.2019.102126

[B45] Ma F. (2014). College English Test: to be abolished or to be polished. J. Lang. Teach. Res. 5, 1176. 10.4304/jltr.5.5.1176-1184

[B46] MacIntyre P. Gregersen T. (2012b). Emotions that facilitate language learning: the positive-broadening power of the imagination. Stud. Second Lang. Learn. Teach. 2, 193–213. 10.14746/ssllt.2012.2.2.4

[B47] MacIntyre P. Gregersen T. Mercer S. (2016). Positive Psychology in SLA. Bristol: Multilingual Matters. 10.21832/9781783095360

[B48] MacIntyre P. D. Gregersen T. (2012a). Affect: the role of language anxiety and other emotions in language learning, in Language Learning Psychology: Research, Theory and Pedagogy, eds Mercer S. Ryan S. Williams M. (Basingstoke: Palgrave), 103–118. 10.1057/9781137032829_8

[B49] MacIntyre P. D. Gregersen T. Mercer S. (2019). Setting an agenda for positive psychology in SLA: theory, practice, and research. Mod. Lang. J. 103, 262–274. 10.1111/modl.12544

[B50] Macnamara B. N. Hambrick D. Z. Oswald F. L. (2014). Deliberate practice and performance in music, games, sports, education, and professions: a meta-analysis. Psychol. Sci. 25, 1608–1618. 10.1177/0956797614535810 24986855

[B51] Mercer S. Ryan S. (2010). A mindset for EFL: learners' beliefs about the role of natural talent. ELT J. 64, 436–444. 10.1093/elt/ccp083

[B52] Mouratidis A. Michou A. Vassiou A. (2017). Adolescents' autonomous functioning and implicit theories of ability as predictors of their school achievement and week-to-week study regulation and well-being. Contemp. Educ. Psychol. 48, 56–66. 10.1016/j.cedpsych.2016.09.001

[B53] Paunesku D. Goldman D. Dweck C. (2011). East Renfrewshire Growth Mindset Study. Glasgow: The Centre for Confidence and Well-being.

[B54] Pekrun R. (2006). The control-value theory of achievement emotions: assumptions, corollaries, and implications for educational research and practice. Educ. Psychol. Rev. 18, 315–341. 10.1007/s10648-006-9029-922364457

[B55] Pekrun R. Frenzel A. C. Goetz T. Perry R. P. (2007). The control-value theory of achievement emotions: An integrative approach to emotions in education, in Educational Psychology Series. Emotion in Education, eds Schutz P. A. Pekrun R. (San Diego, CA: Elsevier Academic Press), 13–36. 10.1016/b978-012372545-5/50003-4

[B56] Peng J.-E. (2019). The roles of multimodal pedagogic effects and classroom environment in willingness to communicate in English. System 82, 161–173. 10.1016/j.system.2019.04.006

[B57] Perkins-Gough D. (2013). The significance of grit: a conversation with Angela Lee Duckworth. Educ. Leadersh. 71, 14–20.

[B58] Piniel K. Albert Á. (2018). Advanced learners' foreign language-related emotions across the four skills. Stud. Second Lang. Learn. Teach. 8, 127–147. 10.14746/ssllt.2018.8.1.6

[B59] Rattan A. Savani K. Chugh D. Dweck C. S. (2015). Leveraging mindsets to promote academic achievement: policy recommendations. Perspect. Psychol. Sci. 10, 721–726. 10.1177/1745691615599383 26581725

[B60] Romero C. Paunesku D. Dweck C. (2010). Preliminary report: Crittenden middle school growth mindset study. Project for Education Research That Scales.

[B61] Rui Y. Muthikrishnan P. (2019). Growth mindset and students' perception of their English language teachers' feedback as predictors of language proficiency of the EFL learners. Asian EFL J. 3, 32–60.

[B62] Ryan S. Mercer S. (2012). Implicit theories: Language learning mindsets, in Psychology for Language Learning: Insights from Research, Theory and Practice, eds Mercer S. Ryan S. Williams M. (New York, NY: Palgrave Macmillan), 74–89. 10.1057/9781137032829_6

[B63] Seligman M. E. Csikszentmihalyi M. (2000). Positive psychology: an introduction. Am. Psychol. 55, 5–14. 10.1037/0003-066X.55.1.5 11392865

[B64] Shao K. Nicholson L. J. Kutuk G. Lei F. (2020). Emotions and instructed language learning: proposing a second language emotions and positive psychology model. Front. Psychol. 11, 2142. 10.3389/fpsyg.2020.02142 32982876 PMC7477346

[B65] Sisk V. F. Burgoyne A. P. Sun J. Butler J. L. Macnamara B. N. (2018). To what extent and under which circumstances are growth mind-sets important to academic achievement? Two meta-analyses. Psychol. Sci. 29, 549–571. 10.1177/0956797617739704 29505339

[B66] Sudina E. Brown J. Datzman B. Oki Y. Song K. Cavanaugh R.. (2020). Language-specific grit: exploring psychometric properties, predictive validity, and differences across contexts. Innov. Lang. Learn. Teach. 15, 1–18. 10.1080/17501229.2020.1802468

[B67] Sudina E. Plonsky L. (2021). Language learning grit, achievement, and anxiety among L2 and L3 learners in Russia. ITL-Int. J. Appl. Linguist. 172, 161–198. 10.1075/itl.20001.sud

[B68] Tang X. Wang M.-T. Guo J. Salmela-Aro K. (2019). Building grit: the longitudinal pathways between mindset, commitment, grit, and academic outcomes. J. Youth Adolesc. 48, 850–863. 10.1007/s10964-019-00998-0 30788767

[B69] Tarbetsky A. L. Collie R. J. Martin A. J. (2016). The role of implicit theories of intelligence and ability in predicting achievement for Indigenous (Aboriginal) Australian students. Contemp. Educ. Psychol. 47, 61–71. 10.1016/j.cedpsych.2016.01.002

[B70] Teimouri Y. Plonsky L. Tabandeh F. (2020). L2 grit: passion and perseverance for second-language learning. Lang. Teach. Res. 10.1177/1362168820921895

[B71] Usher E. L. Li C. R. Butz A. R. Rojas J. P. (2019). Perseverant grit and self-efficacy: are both essential for children's academic success? J. Educ. Psychol. 111, 877. 10.1037/edu0000324

[B72] Wang D. Yuan F. Wang Y. (2020). Growth mindset and academic achievement in Chinese adolescents: a moderated mediation model of reasoning ability and self-affirmation. Curr. Psychol. 41, 1–10. 10.1007/s12144-019-00597-z

[B73] Wang H. Peng A. Patterson M. M. (2021). The roles of class social climate, language mindset, and emotions in predicting willingness to communicate in a foreign language. System 99, 102529. 10.1016/j.system.2021.102529

[B74] Wang R. Sidhu G. K. Muthukrishnan P. (in press). Sustaining quality EFL students' English language performance through achievement goal orientations growth mindset. J. Posit. Sch. Psychol.

[B75] Wang Y. Derakhshan A. Zhang L. J. (2021). Researching and practicing positive psychology in second/foreign language learning and teaching: the past, current status and future directions. Front. Psychol. 12, 731721. 10.3389/fpsyg.2021.731721 34489835 PMC8417049

[B76] Wei H. Gao K. Wang W. (2019). Understanding the relationship between grit and foreign language performance among middle school students: the roles of foreign language enjoyment and classroom environment. Front. Psychol. 10, 1508. 10.3389/fpsyg.2019.01508 31333541 PMC6624730

[B77] Xiao Y. Carless D. R. (2013). Illustrating students' perceptions of English language assessment: voices from China. RELC J. 44, 319–340. 10.1177/0033688213500595

[B78] Yang P. (2021). Exploring the relationship between Chinese EFL students' grit, well-being, and classroom enjoyment. Front. Psychol. 12, 762945. 10.3389/fpsyg.2021.762945 34777167 PMC8586070

[B79] Yeager D. S. Dweck C. S. (2012). Mindsets that promote resilience: when students believe that personal characteristics can be developed. Educ. Psychol. 47, 302–314. 10.1080/00461520.2012.722805

[B80] Zeng G. Hou H. Peng K. (2016). Effect of growth mindset on school engagement and psychological well-being of Chinese primary and middle school students: the mediating role of resilience. Front. Psychol. 7, 1873. 10.3389/fpsyg.2016.01873 28018251 PMC5147462

[B81] Zhang H. Dai Y. Wang Y. (2020). Motivation and second foreign language proficiency: the mediating role of Foreign Language Enjoyment. Sustainability 12, 1302. 10.3390/su12041302

[B82] Zhang L. Chen L. (2015). The evaluation of College English Test Band-4: data analysis on the basis of the classical measurement theory and Rasch model. Contemp. Foreign Lang. Stud. 10, 41–47.

