# Supplementary material for: Relationship Between Growth Mindset and English Language Performance Among Chinese EFL University Students: The Mediating Roles of Grit and Foreign Language Enjoyment
Source: Front Psychol. 2022 Jul 7;13:935506. doi: 10.3389/fpsyg.2022.935506 (PMC9302586; doi:10.3389/fpsyg.2022.935506)
Supplement: Supplementary file 1 [file Data_Sheet_1.doc]

**Appendix A Language Mindsets Inventory**

| **Item**  **Label** | **Sub-Construct/Item** |
| --- | --- |
|  | **General language intelligence beliefs** |
| GM1 | No matter who you are, you can significantly improve your language intelligence level. |
| GM2 | You can always substantially improve your language intelligence. |
| GM3 | No matter how much language intelligence you have, you can always improve it quite a bit. |
|  | **Second language aptitude beliefs** |
| GM4 | You can always improve your foreign language ability. |
| GM5 | In learning a foreign language, if you work hard at it, you will always get better. |
| GM6 | How good you are at using a foreign language will always improve if you really work at it. |
|  | **Age sensitivity beliefs about language learning** |
| GM7 | Everyone could do well in foreign language if they try hard, whether they are young or old. |
| GM8 | How well a person learns a foreign language does not depend on age; anyone who works hard can be a fluent speaker in that language. |
| GM9 | Regardless of the age at which they start, people can learn another language well. |

**Appendix B L2 Grit Scale**

| **Item**  **Label** | **Sub-Construct/Item** |
| --- | --- |
|  | **Perseverance of effort** |
| GR1 | I am a diligent English language learner. |
| GR2 | When it comes to English, I am a hard-working learner. |
| GR3 | Now that I have decided to learn English, nothing can prevent me from reaching this goal. |
| GR4 | I will not allow anything to stop me from my progress in learning English. |
| GR5 | I put much time and effort into improving my English language weaknesses. |
|  | **Consistency of interests** |
| GR6 | I think I have lost my interest in learning English. |
| GR7 | I am not as interested in learning English as I used to be. |
| GR8 | I was obsessed with learning English in the past but have lost interest recently. |

**Appendix C English Classroom Enjoyment Scale**

| **Item**  **Label** | **Sub-Construct/Item** |
| --- | --- |
|  | **Enjoyment of teacher support** |
| FLE1 | The teacher is friendly. |
| FLE2 | The teacher is encouraging. |
| FLE3 | The teacher is supportive. |
|  | **Enjoyment of English language learning** |
| FLE4 | I feel as though I’m a different person during the FL class. |
| FLE5 | I’m a worthy member of the FL class. |
| FLE6 | I can be creative. |
| FLE7 | I don’t get bored. |
| FLE8 | In class, I feel proud of my accomplishments. |
| FLE9 | I’ve learned interesting things. |
| FLE10 | It’s fun. |
| FLE11 | I learned to express myself better in the English learning. |
|  | **Enjoyment of student support** |
| FLE12 | We have common ‘legends’, such as running jokes. |
| FLE13 | We form a tight group. |
| FLE14 | The peers are nice. |
| FLE15 | We laugh a lot. |
